# Neighbourhood-level socio-environmental factors and incidence of first episode psychosis by place at onset in rural Ireland: The Cavan–Monaghan First Episode Psychosis Study [CAMFEPS]^☆^

**DOI:** 10.1016/j.schres.2013.11.019

**Published:** 2014-01

**Authors:** Sami Omer, James B. Kirkbride, Dennis G. Pringle, Vincent Russell, Eadbhard O'Callaghan, John L. Waddington

**Affiliations:** a Department of Psychiatry, College of Medicine, University of Dammam, Saudi Arabia; b Cavan–Monaghan Mental Health Service, Cavan Hospital, Cavan, Ireland; c Molecular & Cellular Therapeutics and 3U Partnership, Royal College of Surgeons in Ireland, Dublin, Ireland; d Department of Psychiatry, University of Cambridge, Cambridge, UK; e Department of Geography and 3U Partnership, National University of Ireland Maynooth, Maynooth, Ireland; f Department of Psychiatry and 3U Partnership, Royal College of Surgeons in Ireland, Beaumont Hospital, Dublin, Ireland; g DETECT Early Psychosis Service, Blackrock, Co. Dublin, Ireland

**Keywords:** First episode psychosis, Place at onset, Rurality, Environmental factors, Social context

## Abstract

**Background:**

Little is known about associations between the social environment and risk for psychosis within rural settings. This study sought to investigate whether such associations exist within a rural context using a prospective dataset of unusual epidemiological completeness.

**Method:**

Using the Cavan–Monaghan First Episode Psychosis Study database of people aged 16 years and older, both ecological analyses and multilevel modelling were applied to investigate associations between incidence of psychosis by place at onset and socio-environmental risk factors of material deprivation, social fragmentation and urban–rural classification across electoral divisions.

**Results:**

The primary finding was an association between more deprived social contexts and higher rates of psychotic disorder, after adjustment for age and sex [all psychoses: incidence rate ratio (IRR) = 1.12, 95% CI (1.03–1.23)].

**Conclusions:**

These findings support an association between adverse socio-environmental factors and increase in risk for psychosis by place at onset within a predominantly rural environment. This study suggests that social environmental characteristics may have an impact on risk across the urban–rural gradient.

## 1 Introduction

The past two decades have witnessed a revival of interest in the role of the social environment in the aetiology of psychotic disorders (Allardyce and Boydell, 2006, Cantor-Graae, 2007, Kirkbride et al., 2007). Socio-environmental risk factors are generally studied at two levels: (1) area-based (contextual) characteristics such as deprivation, social fragmentation and more recently social capital, and (2) individual-level (compositional) factors such as ethnicity, social class, social adversity and cannabis use. A classic example of contextual research is the seminal study by Faris and Dunham in Chicago in the 1930s (Faris and Dunham, 1939). Using first-admission data from psychiatric hospitals over a 12-year period, higher rates of schizophrenia were observed in inner city areas characterised by greater levels of social disorganisation and residential mobility; conversely, rates of ‘manic-depressive’ psychosis (i.e. bipolar disorder) appeared to follow a more random distribution. More recently, several studies have replicated the findings of Faris and Dunham: measures of social fragmentation, including residential mobility and the proportion of single and divorced people in the neighbourhood, were associated with high rates of psychosis (van Os et al., 2000, Silver et al., 2002); similarly, this pattern was evident when a composite measure (the social fragmentation index) was used (Allardyce et al., 2005).

Interactions with neighbourhood-level socio-environmental risk factors appear to be strongest in urban settings (Thornicroft et al., 1993, Allardyce et al., 2005, Zammit et al., 2010). Thus, it has been argued that neighbourhood-level variables may be responsible for differential rates of psychosis between urban and rural environments (Allardyce et al., 2005, Zammit et al., 2010). In Ireland, differential associations between urban and rural areas were found between neighbourhood-level characteristics and rates of self-harm and forensic admissions (O'Neill et al., 2005, Corcoran et al., 2007). The body of literature on contextual research comes from urban settings, with rural areas featuring mainly in urban–rural comparisons. Little is known about associations between the social environment and rates of psychosis within rural settings. In this study, we set out to investigate whether such associations exist within a wholly rural context in Ireland using a dataset of unusual epidemiological completeness.

## 2 Method

### 2.1 Study cohort

Subjects were participants in the Cavan–Monaghan First Episode Psychosis Study (CAMFEPS). This is a prospective study that seeks the closest approximation to identification of ‘all’ incident cases presenting with a first episode of any psychotic disorder in two rural counties in Ireland, Cavan and Monaghan, since 1995, as described previously in detail (Baldwin et al., 2005, Owoeye et al., 2013, Kingston et al., 2013).

In outline, the study involves the following ascertainment procedures: (a) cases identified from all treatment teams in the catchment areas, (b) cases from the catchment areas who present privately to St. Patrick's Hospital or St. John of God Hospital, Dublin, which together account for > 98% of all national private psychiatric admissions, and (c) cases from the catchment areas having forensic admission to the Central Mental Hospital, Dublin. The primary criterion for entry to the study is a first lifetime episode of any psychotic illness at age 16 or above, with no upper age cut-off. DSM-IV diagnosis is made at inception, together with psychopathological and cognitive assessments, reported elsewhere (Owoeye et al., 2013, Kingston et al., 2013), with repeat DSM-IV diagnosis made at 6 months; there are no exclusion criteria other than a previously treated episode of psychosis or psychosis occurring with a prior, overriding diagnosis of gross neurodegenerative disease. This study was approved by the Research Ethics Committees of (initially) the North Eastern Health Board and (subsequent to reorganisation) the Health Service Executive Dublin North East Area, St. Patrick's Hospital, St. John of God Hospital and the Central Mental Hospital, to include (a) subjects giving informed consent to formal assessment and (b) obtaining diagnostic/demographic information from case notes/treating teams for subjects declining formal assessment.

Residence at onset was defined as each subject's domestic location over the 3-month period immediately prior to first presentation with a psychotic illness. For subjects with more than one address in the study area over this period, the address at which he or she was living for more than 50% of the time was applied. Subjects with a second address outside the study area were included only if they were living for more than 50% of the time in Cavan–Monaghan.

### 2.2 Setting

Cavan and Monaghan are two contiguous counties with a population of 109,139 [55,821 males and 53,318 females] at the 2002 census. The region is predominantly rural, consisting of dispersed farms with a scatter of villages and small towns, in the absence of any major urban areas (Central Statistics Office, 2003). The largest towns are the county towns, Cavan and Monaghan, with populations of 5572 and 5557 respectively in 2002. Only one other town had a population of more than 3000 [Carrickmacross, population 3614]. Both counties are ethnically homogeneous, with the vast majority of the population being white Irish. The study is based within Cavan–Monaghan Mental Health Service, a community-based service model comprising two community mental health teams, including home-based treatment teams, a specialist service for the elderly and a community rehabilitation team. Central to the delivery of health services in this model is the use of home-based treatment as an alternative to hospital admission (McCauley et al., 2003, Iqbal et al., 2012). Electoral divisions (EDs) constitute the smallest administrative sub-regions below county level for which census population data are available. The study region contains a total of 155 EDs having a population mean per ED of 697 in 2002 (Central Statistics Office, 2003).

### 2.3 Neighbourhood-level characteristics

ED-based measures were calculated using information from the 2002 census (Central Statistics Office, 2003); this census was closest to the midpoint of the present study (1995–2007).

#### 2.3.1 Material deprivation

Material deprivation was quantified using a deprivation index, similar to the Carstairs (Carstairs and Morris, 1991) and Townsend (Townsend et al., 1988) indices often used in the UK, that was developed by the Small Area Health Research Unit (SAHRU) in Trinity College Dublin (Kelly and Teljeur, 2004). The material deprivation index has been previously used in a variety of contexts, including studies of the availability of psychiatric services (O'Keane et al., 2004), forensic admissions (O'Neill et al., 2005), benzodiazepine consumption (Quigley et al., 2006) and self-harm (Corcoran et al., 2007). This index was constructed for each ED by applying principal components analysis to a combination of selected census-based indicators, including unemployment, social class, type of house tenure and car ownership. EDs are divided into ten categories on an ordinal scale, with 1 being least deprived and 10 most deprived. For the present analyses, these were collapsed into five categories [1 = 1 & 2; 2 = 3 & 4; 3 = 5 & 6; 4 = 7 & 8; 5 = 9 & 10]. Mean deprivation scores and standard deviations (SDs) for the three categories of rurality utilised (see Section 2.3.3 *Urban–rural classification*) were: rural, − 0.37 (0.69); village, 0.52 (0.82); and town, 1.80 (0.67).

#### 2.3.2 Social fragmentation

The social fragmentation index (SFI) was developed for a study of suicide in London (Congdon, 1996). We calculated SFI by adding z scores of four census variables for each ED: 1) non-married adults, 2) single-person households, 3) population turnover and 4) private renting. For the present analyses, the index was collapsed into four categories, created by quartiles, with 1 being least socially fragmented and 4 most socially fragmented. Mean fragmentation scores and standard deviations (SDs) for the three categories of rurality utilised (see Section 2.3.3 *Urban–rural classification*) were: rural, − 0.59 (1.96); village, 1.90 (1.36); and town, 4.96 (2.74).

#### 2.3.3 Urban–rural classification

This classification, developed by SAHRU for health services research at the small area level in Ireland, combines multiple variables, including population density, settlement size and proximity to urban centres (Teljeur and Kelly, 2008); EDs are divided into six categories on an ordinal scale, with 1 being most rural and 6 most urban. For ecological analyses, the urban–rural classification (URC) was collapsed into a three-category variable: URC3 (1 = rural; 2 = village, 3 = town). For multilevel analyses, both URC3 and URC2 (1 = rural, 2 = village & town) were used.

### 2.4 Statistical analysis

We adopted two complementary approaches to data analysis:

First, in accordance with previous literature (Allardyce et al., 2005, Abas et al., 2006, O'Reilly et al., 2008), we aggregated EDs according to neighbourhood-level indices (deprivation scores, fragmentation quartiles and rurality categories), ignoring spatial contiguity. Age-standardised incidence rates (SIRs) were calculated for each category and rate ratios (RRs), with 95% confidence intervals (95% CIs) and associated probabilities, were obtained using category 1 for each neighbourhood-level characteristic as the reference category.

Second, a multilevel Poisson regression model (XTPOISSON command, Stata version 11.1; Kirkbride et al., 2007, Kirkbride et al., 2008) was applied, by which area-based measures were treated as continuous z-standardised (deprivation, SFI) or categorical (URC) variables. This approach allowed us to adjust more fully for potential confounding by individual- and neighbourhood-level variables. Incidence of psychosis was modelled, with variation in incidence quantified by fitting normally distributed random effects at the ED level (i.e. a random intercepts model). Neighbourhood-level characteristics were entered as fixed effects, using a forward-fitting modelling strategy. The natural logarithm of the denominator population, adjusted for the 12-year study period, was entered as an offset term in these models. Significance testing of fixed effects and their interactions was conducted using likelihood ratio tests (LRTs). To inspect for the possibility of over-dispersion in our models at the ED level (more zero counts of cases than expected under a Poisson distribution), we re-fitted our final models under a zero-inflated-Poisson (ZIP) regression, using a Vuong test to test for evidence of over-dispersion. In all analyses a multilevel Poisson model was found to perform satisfactorily (data available on request).

## 3 Results

During the first 12 years of the present study, May 1995–April 2007, CAMFEPS incepted 336 cases of any DSM-IV psychotic illness. Cases of non-functional psychosis [i.e. substance-induced psychosis or psychosis due to a general medical condition], those with no fixed address and those whose onset of illness was outside of Cavan–Monaghan were excluded from the study. As genetic risk in first-degree relatives is, in general, considerably larger than risk associated with environmental factors, a conservative approach was adopted to control for genetic relatedness as a potential confound in evaluating putative environmental factors related to small area variation in rate: in multiply affected families, only the first-born was included (Youssef et al., 1999, Scully et al., 2004). Thus, the total number of cases of functional psychotic illness [hereafter ‘all psychoses’] included in this analysis was 255 [144 males, 111 females]. Cases were further subdivided into two broad diagnostic categories: a) ‘non-affective psychoses’ [primarily schizophrenia and schizoaffective disorder: *n* = 132; 83 males, 49 females]; and b) ‘affective psychoses’ [bipolar disorder and major depressive disorder with psychotic features: *n* = 123; 61 males, 62 females].

### 3.1 Ecological analysis

#### 3.1.1 Material deprivation

For ‘all psychoses’, increase in level of deprivation was associated ordinally with increase in incidence rate among men but not women (Table 1). When ‘non-affective psychoses’ and ‘affective psychoses’ were considered separately, similar but less robust patterns were found (data available on request).

**Table 1:** Age-standardised incidence rates (SIRs, with 95% CIs) per 100,000 and rate ratios (RRs, with 95% CIs) for all psychoses by material deprivation index.

Category	Men						Women					
	SIR	(95% CI)	RR	(95% CI)	*n*	Population	SIR	(95% CI)	RR	(95% CI)	*n*	Population
1	16.63	(13.45–19.80)	1	–	11	5616	19.55	(16.04–23.07)	1	–	13	5532
2	19.77	(16.31–23.23)	1.19	(0.92–1.53)	21	8944	12.28	(9.47–15.08)	0.63	(0.47–0.83)⁎	12	8152
3	20.00	(16.52–23.47)	1.20	(0.93–1.55)	33	14,019	17.18	(13.88–20.49)	0.88	(0.68–1.14)	26	12,821
4	21.41	(17.81–25.00)	1.29	(1.00–1.66)⁎	33	12,849	17.01	(13.72–20.30)	0.87	(0.67–1.13)	25	12,273
5	26.31	(22.33–30.29)	1.58	(1.25–2.01)⁎	46	13,737	19.60	(16.08–23.12)	1.00	(0.78–1.29)	35	14,008

Category 1: least deprived (reference); category 5: most deprived.

⁎ *p* < 0.05.

#### 3.1.2 Social fragmentation

For ‘all psychoses’, the highest rate of psychosis among women was in the most socially fragmented areas; this pattern was evident for both ‘non-affective psychoses’ [RR 1.78 (1.18–2.67)] and ‘affective psychoses’ [RR 1.69 (1.20–2.37)]. There were no significant associations between rate of psychosis and social fragmentation among men (Table 2).

**Table 2:** Age-standardised incidence rates (SIRs, with 95% CIs) per 100,000 and rate ratios (RRs, with 95% CIs) for all psychoses by social fragmentation index.

Category	Men						Women					
	SIR	CI	RR	CI	*n*	Population	SIR	CI	RR	CI	*n*	Population
1	21.58	(17.97–25.19)	1	–	25	9785	14.10	(11.10–17.10)	1	–	15	8986
2	17.55	(14.28–20.81)	0.81	(0.64–1.04)	21	10,232	11.25	(8.56–13.94)	0.80	(0.59–1.09)	12	9183
3	20.85	(17.30–24.40)	0.97	(0.77–1.22)	36	14,593	12.79	(9.93–15.65)	0.90	(0.67–1.23)	21	14,005
4	24.57	(20.72–28.41)	1.14	(0.91–1.43)	62	20,555	24.29	(20.38–28.21)	1.72	(1.33–2.24)⁎	63	20,612

Category 1: most socially cohesive (reference); category 4: most socially fragmented.

⁎ *p* < 0.05.

#### 3.1.3 Urban/rural classification

For ‘all psychoses’, the highest rates of psychosis among women were in the least rural areas; this pattern was evident for ‘affective psychoses’ [RR 1.42 (1.01–2.01)] but not for ‘non-affective psychoses’. There were no significant associations between rates of psychosis and urban/rural classification among men (Table 3).

**Table 3:** Age-standardised incidence rates (SIRs, with 95% CIs) per 100,000 and rate ratios (RRs, with 95% CIs) for all psychoses by urban–rural classification.

Category	Men						Women					
	SIR	CI	RR	CI	*n*	Population	SIR	CI	RR	CI	*n*	Population
1	21.64	(18.02–25.25)	1	–	90	35,210	15.53	(12.39–18.68)	1	–	59	32,249
2	20.69	(17.15–24.22)	0.96	(0.76–1.21)	19	7512	19.2367	(15.75–22.73)	1.24	(0.95–1.62)	17	7453
3	22.08	(18.43–25.73)	1.02	(0.81–1.29)	35	12,443	20.7117	(17.09–24.33)	1.33	(1.03–1.73)⁎	35	13,084

Category 1: most rural (reference); category 3: least rural.

⁎ *p* < 0.05.

### 3.2 Multilevel analysis

Relationships between individual-level variables, neighbourhood-level variables and incidence of ‘all psychoses’ are shown in Table 4. As expected, risk was highest among the 15–24 age group and declined over subsequent decades until around 65 years of age, after which risk increased slightly; decline in risk with age was less marked among women than among men, in accordance with previous findings (Kirkbride et al., 2006).

**Table 4:** Modelling of individual- and neighbourhood-level socio-environmental risk factors for all psychoses.

Variable	Strata	All subjects: IRR (95% CI)			Men: IRR (95% CI)			Women: IRR (95% CI)		
		Unadjusted	Full	LRT *p*	Unadjusted	Full	LRT *p*	Unadjusted	Full	LRT *p*
*Individual-level variables*										
Age	15–24	1	1	0.001	1	1	0.001	1	1	0.69
	25–34	0.8 (0.5–1.1)	0.8 (0.6–1.1)		0.7 (0.4–1.0)	0.7 (0.4–1.0)		0.8 (0.5–1.5)	1.0 (0.5–1.8)	
	35–44	0.5 (0.4–0.8)	0.5 (0.6–0.8)		0.4 (0.3–0.7)	0.4 (0.3–0.7)		0.7 (0.4–1.3)	0.8 (0.4–1.4)	
	45–54	0.5 (0.3–0.8)	0.5 (0.6–0.8)		0.3 (0.2–0.6)	0.3 (0.2–0.6)		0.8 (0.4–1.5)	0.9 (0.5–1.7)	
	55–64	0.4 (0.3–0.7)	0.4 (0.3–0.7)		0.4 (0.2–0.7)	0.4 (0.2–0.7)		0.5 (0.2–1.1)	0.6 (0.3–1.3)	
	65–74	0.6 (0.4–1.0)	0.6 (0.4–1.0)		0.4 (0.2–0.8)	0.4 (0.2–0.8)		1.0 (0.5–2.0)	1.0 (0.5–1.9)	
	75 +	0.9 (0.5–1.3)	0.9 (0.6–1.4)		0.8 (0.4–1.5)	0.8 (0.4–1.5)		1.1 (0.6–2.2)	1.1 (0.6–2.1)	
Sex	Women vs men	0.8 (0.6, 1.0)	0.8 (0.6, 1.0)	0.08	–	–		–	–	

*Neighbourhood-level variables*										
SFI	1 SD change	1.10 (1.01–1.20)	–	0.60	1.04 (0.92–1.17)	–	0.61	1.17 (1.04–1.32)	–	0.39
Deprivation	1 SD change	1.13 (1.03–1.24)	1.12 (1.03–1.23)	0.01	1.10 (0.97–1.25)	–	0.16	1.18 (1.03–1.36)	1.16 (1.01–1.32)	0.05
URC3	Rural	1	–	0.86	1	–	0.94	1	–	0.76
	Village	1.07 (0.74–1.54)	–		0.97 (0.59–1.60)	–		1.13 (0.66–1.94)	–	
	Town	1.19 (0.90–1.58)	–		1.07 (0.73–1.58)	–		1.36 (0.90–2.07)	–	
URC2	Rural	1	–	0.58	1	–	0.93	1	–	0.79
	Less rural	1.15 (0.89–1.47)	–		1.04 (0.74–1.45)	–		1.28 (0.88–1.85)	–	

IRR, incidence rate ratio (with 95% CI); LRT, likelihood ratio test; *p* value indicates whether a variable improved overall model fit. IRR not reported for variables that did not significantly improve the final model at the *p* < 0.05 threshold of significance.

SFI, social fragmentation index; URC, urban–rural classification; Deprivation, material deprivation index; SD, standard deviation.

In the unadjusted multilevel model, no significant neighbourhood variation (i.e. random effects) in incidence rates was apparent. Despite this, however, we did observe a relationship between increased level of deprivation and higher incidence rates of psychosis in our fully adjusted model (for age and sex); when stratified by sex, this effect was evident only among women. No such association was evident when the sample was restricted to those ages studied typically in first episode samples (15–64 years); a marginal interaction between age group and level of deprivation in the full sample (LRT, *p* = 0.08) suggested further that the association between level of deprivation and risk for psychosis derived primarily from the group aged 65–74 years. When ‘non-affective psychoses’ and ‘affective psychoses’ were analysed separately, no associations between any neighbourhood-level variable and risk for psychosis were evident (data available on request).

## 4 Discussion

To our knowledge, this is the first study to examine associations between neighbourhood-level socio-environmental risk factors and incidence of first episode psychosis within a rural setting. Unlike the majority of other studies, we did not impose any upper age limit and attempted to identify ‘all’ cases presenting with a first episode psychosis so as to incept an epidemiologically representative population across the lifespan. We adopted, and thus were able to compare, two complementary approaches to data analysis. First, by aggregating EDs according to their social characteristics; this is a widely adopted ecological approach (Allardyce et al., 2005, Abas et al., 2006, O'Reilly et al., 2008) that reveals non-linear relationships where they exist and does not depend on any assumptions applicable to multilevel techniques. Second, by applying multilevel modelling; this more incisive approach allows exploration of variation in the incidence of first episode psychosis at more than one level (Kirkbride et al., 2007, Kirkbride et al., 2008). The main findings are considered below.

### 4.1 Material deprivation

Ecological analysis identified a variable association between extent of deprivation and incidence of psychosis, primarily among men; previous ecological studies have demonstrated that the relationship between deprivation and psychosis is not necessarily linear (Croudace et al., 2000, Allardyce and Boydell, 2006). Multilevel modelling revealed an association between extent of deprivation and risk for ‘all psychoses’ for the whole sample, though this effect may have been restricted to older women, beyond the age range considered in most studies of first episode psychosis. These findings suggest that women may be particularly sensitive to deprivation during late rather than early life. Alternatively, they may reflect cumulative exposure to deprivation over the life course in women or, perhaps, stronger social drift for women who go on to develop psychosis later in life as they become more marginalised in rural communities; however, longitudinal data would be necessary to test such hypotheses. Our data suggest that socio-environmental factors influence incidence rates in rural as well as urban communities. Although the impact of such factors may be greater in more urban regions, our results nonetheless hold material import for health service planning, public health and, more tentatively, etiological research.

### 4.2 Social fragmentation and rurality

Ecological analysis revealed an association between extent of social fragmentation and incidence of psychoses, primarily among women. In multilevel analyses, social fragmentation was not associated with the rate of psychosis once material deprivation has been included, though they were highly correlated factors [r^2^ = 0.63, *p* < 0.01]. Whether social fragmentation or material deprivation constitutes the more salient exposure remains a matter of debate but studies conducted in other settings indicate a more robust association between area-level social fragmentation and rate of psychosis, primarily in urban settings (Allardyce et al., 2005, Zammit et al., 2010). As a modest association between social fragmentation and incidence of psychosis was evident here on ecological analysis but not using multilevel modelling, future studies should examine further the extent to which this may reflect lower levels of, or less variability in, social fragmentation in rural compared to urban areas. A previous nation-wide study from Ireland has demonstrated the lowest scores for social fragmentation to be in rural EDs, with the highest scores reported in cities other than Dublin (Corcoran et al., 2007).

While ecological analysis also indicated that women living in the least rural areas of our study were at increased risk for psychosis, this association was not evident in multilevel analyses after taking age and deprivation into account. While there is a strong body of evidence indicating higher rates of psychosis in urban areas (Pedersen and Mortensen, 2001, Harrison et al., 2003, McGrath et al., 2004, Kelly et al., 2010), future studies should examine further the extent to which socio-environmental variation in risk for psychosis may extend beyond the traditional dichotomous urban/rural divide and be subject to gradations within both urban *and* rural areas.

In ecological analyses the associations between incidence of psychosis and neighbourhood indices of social fragmentation and rurality showed a similar profile for affective and non-affective psychoses. This contrasts with findings by Faris and Dunham (1939). While we cannot exclude the possibility of misclassification of cases, we believe this to be unlikely as standardised assessment methods were employed for diagnosis using DSM-IV criteria. While the absence of significant findings using multilevel modelling is cautionary, other ecological studies indicate that the relationship between contextual characteristics and mental illness is not confined to non-affective psychoses and extends to affective disorders (Silver et al., 2002, Curtis et al., 2006).

### 4.3 Gender

Our findings include a number of gender-related associations that were apparent across diagnostic categories, not confined to a single index and evident using either analytical approach. A large population-based case–control study of risk for myocardial infarction in Sweden revealed gender differences in contextual effects of material deprivation and social fragmentation (Stjärne et al., 2004); similarly, gender differences have been reported in ecological studies of self-rated health (Stafford et al., 2005, Kavanagh et al., 2006). Possible explanations for such gender differences are that men and women differ in perception of and exposure and vulnerability to the local environment (Stafford et al., 2005).

### 4.4 Methodological considerations

#### 4.4.1 Strengths

A strength of this study is the epidemiological completeness of the data: the Irish mental health service operates a strict catchment area policy, such that patients presenting to services other than those relating to their home address are re-directed to their catchment area; we were able to ascertain cases who chose to present privately or whose presentation resulted in a forensic admission; we were able to obtain information from case records/treating teams for subjects who declined formal assessment. Therefore, the likelihood of case leakage is considerably reduced. A common methodological challenge for ecological studies is reliance on hospital registers and case record diagnoses as outcome measures; in our study, cases were accrued prospectively and operational diagnostic criteria were used. The ethnic homogeneity and lack of in-migration in the Cavan–Monaghan region allow for examination of neighbourhood-level effects independent of migration, a potential problem for urban-based studies. Multilevel analyses allowed us to quantify the impact of neighbourhood-level factors on incidence rates in our sample. While multilevel models suggested only weak neighbourhood (random) effects, our study was sufficiently powered to detect evidence for an association between material deprivation and psychosis after controlling for individual level covariates. These multilevel models were appropriate for count data and were fitted appropriately, given the possibility of over-dispersion.

#### 4.4.2 Limitations

Our study is subject to a number of limitations common to ecological research. First, we cannot assume that people with first episode psychosis living in neighbourhoods with a given level of a putative risk factor were themselves exposed to that level of risk (the ‘ecological fallacy’). Second, the cross-sectional design of ecological studies precludes assessing the direction of causality in any associations, such that we cannot exclude a role for social drift. A longitudinal study design may help address this issue, including whether social drift may extend to generations beyond cases and their parents (Goldberg and Morrison, 1963). Although every effort was made to identify every new case in the study area over a 12-year period, the total number of cases is not large; caution must therefore be exercised in the interpretation of the results, especially when analysing sub-groups in the total sample (e.g. male/female, affective/non-affective cases). Ascertaining an appropriate denominator in such population-based research also remains a challenge. We did not have data at an individual level on some possible confounders, such as social class. Some of our findings could be mediated by aggregation of individual-level characteristics, though we controlled for two very important factors: age and sex. However, in previous large population studies the effect of neighbourhood-level social fragmentation has remained after controlling for individual-level characteristics (Silver et al., 2002, Zammit et al., 2010). The comparison of two analytical approaches revealed both convergence on certain relationships and some differences in specifics; this emphasises how conclusions drawn can be influenced by the analytical approach adopted.

A potential limitation is differential migration. For example, it is possible that areas of high incidence are related to outmigration of healthy subjects or, alternatively, that areas of low incidence are related to outmigration of those at risk for psychosis. On demographic grounds, over the period during which the present data were collected (1995–2007), such selective migration is unlikely to be so substantive as to generate the present profile of results. However, the absence of information on the mental health of those who may have left the study area is cautionary. A further limitation is the use of census-based data as a measure of area characteristics. Composite measures such as the social fragmentation index are artificial constructs based on indicators whose selection is dictated by availability of census data (Congdon, 2004). It is also possible that census-based indicators such as private renting and single-person households may not capture social fragmentation similarly in urban and rural areas (Allardyce et al., 2005). We attempted to control for genetic relatedness by including only the first-born in multiply affected families, but this may not control adequately for the role of family history in clustering of cases. Finally, the 2002 census may not be truly representative of the social characteristics of Cavan–Monaghan over the 12-year period of the study. To address this, we repeated analyses using data from the 2006 census; this did not materially alter our results (data available on request).

Our primary finding is an association between more deprived social contexts and higher rates of psychotic disorder in a predominantly rural setting, after adjustment for age and sex. However, there was less evidence for such associations than is typically reported in urban settings (Kirkbride et al., 2007). Together, these findings suggest that there may be a continuum of risk of psychosis with socio-environmental risk factors across the rural–urban divide, to include essentially rural environments. However, they suggest that such exposures may have greater impact in more urban settings. Future epidemiological and health services research in rural areas (see, for example, the SEPEA study (Kirkbride et al., 2012)) should take into account potential variations in risk within rural areas and consider gender differences in relation to contextual effects.

## Role of funding source

The Cavan–Monaghan First Episode Psychosis Study [CAMFEPS] was supported by the Stanley Medical Research Institute and Cavan–Monaghan Mental Health Service. J.B. Kirkbride was supported by a Sir Henry Wellcome Research Fellowship from the Wellcome Trust (grant number WT085540).

## Contributors

Sami Omer contributed to the conception and design of the study, and collection, analysis and interpretation of the data, drafted the manuscript and ensured final approval of the version to be published. James Kirkbride contributed to the analysis and interpretation of the data, revising the manuscript and final approval of the version to be published. Dennis Pringle contributed to the analysis and interpretation of the data, revising the manuscript and final approval of the version to be published. Vincent Russell contributed to the conception and design of the study, revising the manuscript and final approval of the version to be published. Eadbhard O'Callaghan contributed to the conception and design of the study; his tragic death precluded giving final approval of the version to be published. John Waddington contributed to the conception and design of the study, collection, analysis and interpretation of the data, revising the manuscript and final approval of the version to be published.

## Conflict of interest

None of the authors have any conflict of interest relating to this manuscript.
